# ﻿A new species of *Physomerinus* Jeannel (Coleoptera, Staphylinidae, Pselaphinae) from Jiulong National Wetland Park, China

**DOI:** 10.3897/zookeys.1153.100073

**Published:** 2023-03-16

**Authors:** Zi-Wei Yin

**Affiliations:** 1 Laboratory of Systematic Entomology, College of Life Sciences, Shanghai Normal University, Shanghai 200234, China Shanghai Normal University Shanghai China

**Keywords:** Ant-loving beetle, Batrisina, Batrisini, East China, new taxon, taxonomy, Zhejiang

## Abstract

*Physomerinusjiulongensis***sp. nov.** is described from a series of overwintering individuals collected in decomposing wood at Jiulong National Wetland Park, East China. The new species is characterized and separated from related congeners by the unique form of the sexually dimorphic maxillary palpi, greatly swollen male metafemora, as well as by the shape of the genitalia of both sexes. A key to, and a distributional map of, *Physomerinus* species occurring in China and on the Ryukyu Islands, Japan is provided.

## ﻿Introduction

The ant-loving beetle genus *Physomerinus* Jeannel, 1952 (Batrisitae: Batrisini) contains 12 species distributed in the Oriental and East Palaearctic regions (Newton 2022). The genus was placed in Jeannel’s fifth division of the Batrisina (Jeannel 1958), or as a member of Nomura’s (1991) ‘*Batrisocenus* complex’ of genera. Species of this genus have the pronotum with distinct median and two lateral longitudinal sulci, and a complete transverse antebasal sulcus, the sides of the pronotum lack spines, each elytron has two basal foveae, and the first visible tergite (IV) is longest. The males possess variously modified metafemora, and often a constricted basal capsule of the aedeagus. The latter indicates a close relationship to the genus *Batriscenaulax* Jeannel, 1958 which includes six species from Japan and China (Nomura 1991; Yin 2022). One major taxonomic problem of *Physomerinus* is that the type species, *P.septemfoveolatus* (Schaufuss L. W., 1877) from Southeast Asia, has a secretory trichome on the anterolateral margin of the scape, and the aedeagus bears a large basal capsule (Jeannel 1952: fig. 42; Nomura pers. com.), which are congruent with the defining features of a group of genera centered on *Batrisiella* Raffray, 1904. A revision of the genus is needed to clarify this problem.

Within *Physomerinus* several species may indeed form monophyletic subgroups by their shared male features. *Physomerinushasegawai* Nomura, 1991 from China (Taiwan) and Japan (Ryukyu Islands) and *P.clavipes* Zhang & Yin, 2022 from China (Guangxi) are linked by the smoothly broadened lateral portions of maxillary palpomeres 4, and greatly swollen metafemora with a large lateral cavity (Nomura 1991, 2010, 2012, 2013; Zhang and Yin 2022).

During December 2022 to January 2023, a team of IZCAS led by Hong-Bin Liang surveyed the beetle fauna of Jiulong National Wetland Park in Zhejiang Province, East China. A series of overwintering pselaphine beetles were collected from decomposing wood along a riverbank and were later sent to me for identification. The result revealed a new species of *Physomerinus* closely related to *P.hasegawai* and *P.clavipes*, which is diagnosed, described, and illustrated in this paper.

## ﻿Material and methods

The holotype of the new species described here is deposited in the Institute of Zoology, Chinese Academy of Science (**IZCAS**), Beijing, China, and the paratypes in IZCAS and the Insect Collection of Shanghai Normal University (**SNUC**), Shanghai, China. The label data of the material are quoted verbatim.

Dissected parts were mounted in Euparal on plastic slides pinned with the specimen. The habitus image of the beetle was taken using a Canon 5D Mark III camera, equipped with a 7.5× Mitutoyo M Plan Apo lens, and two Godox V860III-C TTL Li-Ion flashes were used as the light source. Images of the morphological details were produced using a Canon G9 camera mounted to an Olympus CX31 microscope under reflected or transmitted light. Helicon Focus v. 8.2.0 Pro was used for image stacking. All images were modified and grouped into plates using Adobe Photoshop CC 2020.

Measurements were taken as follows: total body length was measured from the anterior margin of the clypeus to the apex of the abdomen; head length was measured from the anterior margin of the clypeus to the head base, excluding the occipital constriction; head width was measured across the eyes; the length of the pronotum was measured along the midline, the width of the pronotum is its maximum width; the length of the elytra was measured along the suture; the width of the elytra was measured as the maximum width across both elytra; the length of the abdomen is the length of the dorsally exposed part of the abdomen along its midline, the width of the abdomen is its maximum width. Terminology follows Chandler (2001) and Yin (2022). The abdominal tergites and sternites are numbered in Arabic (starting from the first visible segment) and Roman (reflecting true morphological position) numerals, e.g., tergite 1 (IV), or sternite 1 (III). Paired appendages in the description of the new species are treated as singular.

## ﻿Taxonomy

### ﻿ Physomerinus jiulongensis

Taxon classificationAnimaliaColeopteraStaphylinidae

Yin sp. nov.

E4538BFB-BEFF-5E21-A9BD-B0492FDBD0E1

https://zoobank.org/A558FA35-B929-44A5-B490-613DE3074175

**Figure 1.**
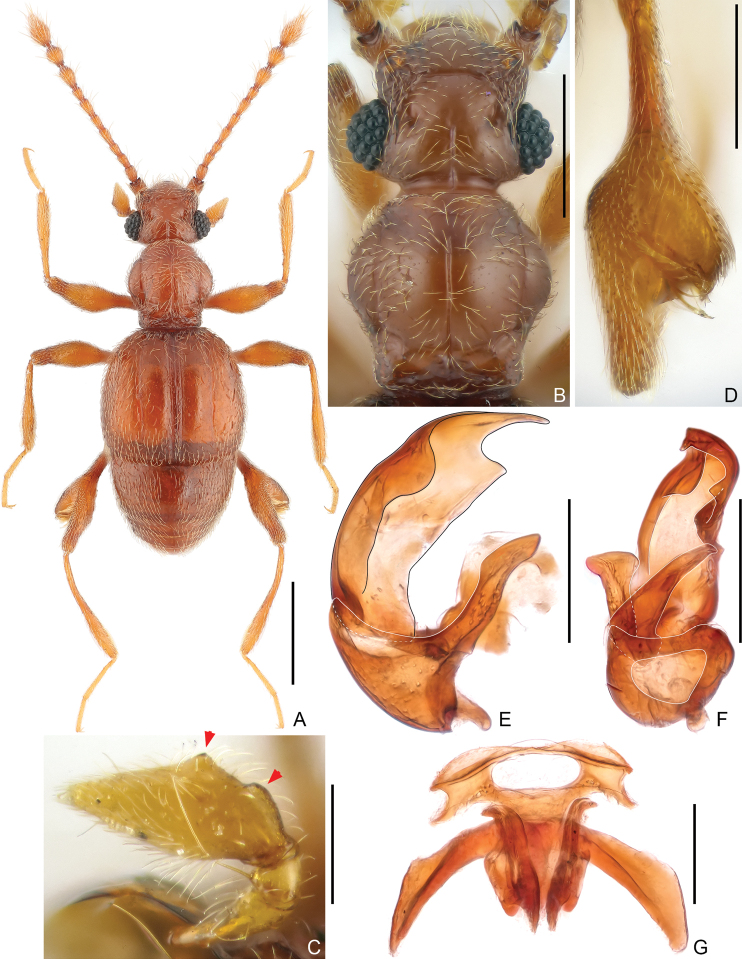
Morphology of *Physomerinusjiulongensis* sp. nov. (**A–F** male **G** female) **A** habitus **B** head and pronotum **C** maxillary palpus **D** metafemur **E, F** aedeagus, lateral (**E**) and ventral (**F**) **G** genital complex. Scale bars: 0.5 mm (**A**); 0.3 mm (**B**); 0.1 mm (**C, E, F, G**); 0.2 mm (**D**).

**Figure 2.**
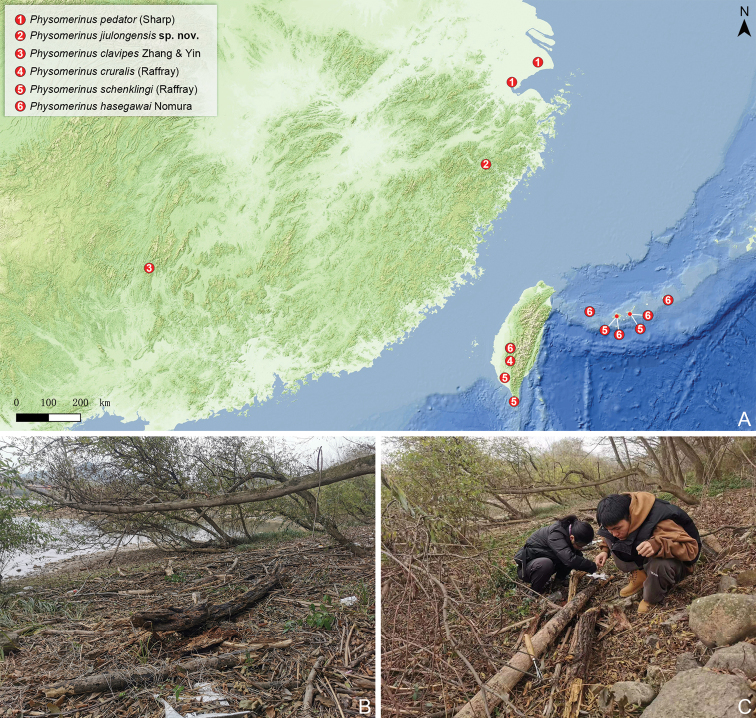
Distribution and habitat of *Physomerinus***A** map showing the distribution of *Physomerinus* species occurring in China and on the Ryukyu Islands, Japan **B, C** collecting environment (**B**) and habitat (**C**) of *P.jiulongensis* sp. nov. in Jiulong National Wetland Park, Zhejiang, China. Yu-Yao Qin (left) and Kai Wang (right) were searching for pselaphines from decomposing wood.

Figs 1 , 2 Chinese common name: 九龙奇腿蚁甲

#### Type material

**(25 exx.). *Holotype*: China**: ♂, ‘China: Zhejiang, Lishui, Bihu To., Hongyu Vill., Jiulong Wetland Park, 28.38479°N, 119.8247°E, 60 m, 31.xii.2022, wood, Liang, Qin, Wang leg. [丽水九龙湿地公园, 梁红斌, 秦雨瑶, 王凯 采]’ (IZCAS). ***Paratypes*: China**: 9 ♂♂, 8 ♀♀, same collecting data as for holotype; 4 ♂♂, 3 ♀♀, same collecting data as for holotype, except ‘4.i.2023’ (4 exx. in IZCAS, 20 exx. in SNUC).

#### Diagnosis.

**Male.** Body length approximately 1.8 mm. Head subtruncate at base; vertex with transverse sulcus posterior antennal tubercles, with short mediobasal carina, vertexal foveae small and asetose; antenna relatively long, antennomeres each elongate, lacking modification; maxillary palpomere 4 protuberant on lateral margin. Discal stria of elytron extending posteriorly to near posterior margin. Metafemur greatly swollen and with large cavity on lateral side. Abdomen dorsally with tergite 1 (IV) longer than 2–4 (V–VII) combined in dorsal view, simple. Aedeagus strongly asymmetric, ventral stalk much shorter than dorsal lobe. **Female.** Body length slightly over 1.8 mm, maxillary palpus and metafemur lacking modification, genital complex as in Fig. 1G.

#### Description.

**Male.** Body (Fig. 1A) length 1.77–1.81 mm; color reddish-brown, tarsi and mouthparts lighter in color. Dorsal surface of body covered with relatively short pubescence.

Head (Fig. 1B) subtruncate at base, much wider than long, length 0.35–0.36 mm, width across eyes 0.40–0.43 mm; vertex finely punctate, smoothly convex, with small, asetose and broadly separated vertexal foveae (dorsal tentorial pits), with short, straight transverse sulcus posterior antennal tubercles, mediobasal carina extending posteriorly to occipital constriction and anteriorly to level of slightly posterior midlength of eyes, antennal tubercle moderately raised, surrounding area roughly punctate; frons broadly and shallowly impressed medially, confluent with clypeus; clypeus with smooth surface, its anterior margin carinate and moderately raised; ocular-mandibular carina complete. Venter with tiny gular foveae (posterior tentorial pits) originating from shared transverse slit, with distinct median carina extending from foveae anteriorly to mouthparts. Compound eyes greatly prominent, composed of approximately 35 large ommatidia. Antenna 0.97–1.01 mm long, lacking modification; antennomere 1 thick, subcylindrical, 2–7 each slightly elongate, successively longer, 8 shortest, slightly elongate, enlarged 9–11 forming distinct club, 9 and 10 of subequal size, each moderately expanded on mesal margin, 11 largest, 1.7× as long as 10, fusiform, truncate at base.

Pronotum (Fig. 1B) slightly longer than wide, length 0.41–0.44 mm, width 0.40–0.42 mm, widest at middle; lateral margins rounded; disc moderately convex, finely punctate, with median longitudinal sulcus much shorter than semicircular lateral sulci in dorsal view; lacking median antebasal fovea, with complete, deep transverse antebasal sulcus connecting lateral antebasal foveae, with small, blunt antebasal tubercles; outer and inner pair of basolateral foveae small. Prosternum with anterior part as long as coxal part, with small lateral procoxal foveae; hypomeral ridge complete, with lateral antebasal hypomeral pit; margin of coxal cavity thinly carinate.

Elytra much wider than long, length 0.61–0.62 mm, width 0.69–0.72 mm; each elytron with two large, widely separated basal foveae, lacking subbasal fovea; humeral protuberance small, acute; discal stria long, extending from outer basal fovea to approximately apical 4/5 of elytral length; subhumeral fovea small, carinate marginal stria extending posteriorly from fovea to apex of elytron. Metathoracic wings fully developed.

Mesoventrite short, demarcated from metaventrite by ridged anterior edges of impressed areas where large, setose lateral mesocoxal foveae situated at mesal portions of impression, with pair of admesal carinae; setose median mesoventral foveae broadly separated, lateral mesoventral foveae large and setose, forked internally. Metaventrite moderately impressed at middle, with pair of setose lateral metaventral foveae, posterior margin with small and narrow split at middle.

Legs elongate; mesotrochanter with tiny, indistinct protuberance on ventral margin; metafemur greatly swollen laterally and with large cavity (Fig. 1D), dorsal side with short sensory setae, with tufts of setae along mesal and posterior margin of cavity.

Abdomen much narrower than elytra, widest at basolateral margins of tergite 1 (IV), length 0.44–0.52 mm, width 0.62–0.65 mm; lacking modification. Tergite 1 (IV) in dorsal view longer than 2–4 (V–VII) combined; setose basal sulcus separated by mediobasal and one pair of basolateral foveae, with pair of thin, triangular discal carinae; tergites 2 and 3 (V and VI) each short, lacking fovea, 4 (VII) as long as 2 and 3 combined along middle, with one pair of small basolateral foveae, 5 (VIII) semicircular, posterior margin roundly emarginate at middle. Sternite 2 (IV) with one pair of mediobasal and one pair of basolateral foveae, and large basolateral sockets, with one pair of short and one pair of long lateral carinae; midlength of sternite 2 shorter than 3 (V) and 4 (VI) combined, 3–5 (VII) each short, successively shorter, lacking fovea, 6 (VIII) greatly transverse, posterior margin roundly convex at middle, sternite 7 (IX) membranous, indistinct.

Aedeagus (Fig. 1E, F) 0.21 mm long, strongly asymmetric; median lobe with constricted basal capsule and sub-triangular foramen, with long basoventral projection, ventral stalk short, in lateral view curved and narrowing towards apex; dorsal lobe much longer than ventral stalk, broad, sides incurved, with narrowed apex; parameres fused to form broad, semi-sclerotized plate.

**Female.** Similar to male in external morphology; antenna slightly shorter; maxillary palpus and metafemur lacking modification; each compound eye composed of approximately 30 ommatidia; humeral protuberance small and weak; metathoracic wings reduced. Measurements (as for male): body length 1.83–1.85 mm; length/width of head 0.35–0.37/0.41–0.43 mm, pronotum 0.40–0.43/0.40–0.43 mm, elytra 0.54–0.57/0.66–0.71 mm; abdomen 0.50–0.54/0.65–0.67 mm; length of antenna 0.92–0.96 mm; maximum width of genital complex (Fig. 1G) 0.29 mm, genital plate much wider than sternite 9, lateral arms broadened distally.

#### Comparative notes.

This species shares with *P.hasegawai* from Taiwan and Japan and *P.clavipes* from Guangxi the sexually dimorphic fourth segments of the maxillary palpi and greatly swollen metafemora of the male, and together these species may form a monophyletic group. The male of *Physomerinusjiulongensis* sp. nov. can be readily separated by the presence of two protuberances on the lateral margins of maxillary palpomeres 4, and short ventral lobe of the aedeagus. In contrast, both *P.hasegawai* and *P.clavipes* have smoothly swollen lateral margins of maxillary palpomeres 4, and the ventral stalks of the aedeagi are as long as or slightly longer than the dorsal lobes.

#### Distribution.

East China: Zhejiang (Fig. 2A).

#### Bionomics.

All overwintering adults were collected in decomposing wood near a river bank (Fig. 2B, C).

#### Etymology.

The new species is named after its type locality, i.e., Jiulong National Wetland Park.

### ﻿Key to males of *Physomerinus* species from China and the Ryukyu Islands, Japan

**Table:** 

1	Lateral margin of maxillary palpomere 4 smoothly expanded or protuberant	**2**
–	Maxillary palpomere 4 fusiform; lateral margin not expanded and lacking protuberance	**4**
2	Lateral margin of maxillary palpomere 4 smoothly expanded; aedeagus with ventral stalk as long as or slightly longer than dorsal lobe	**3**
–	Lateral margin of maxillary palpomere 4 with two protuberances (Fig. 1C); aedeagus with ventral stalk much shorter than dorsal lobe (Fig. 1E, F) (Zhejiang)	***P.jiulongensis* sp. nov.**
3	Hind legs relatively much longer (Zhang and Yin 2022: fig. 2A); aedeagus with ventral stalk slightly longer than dorsal lobe, and expanded at middle in dorso-ventral view (Zhang and Yin 2022: fig. 2F) (Guangxi)	***P.clavipes* Zhang & Yin**
–	Hind legs relatively much shorter; aedeagus with ventral stalk as long as dorsal lobe, and slightly broadened from base toward apical portion in dorso-ventral view (Nomura 1991: fig. 134G) (Taiwan; Ryukyu Islands)	***P.hasegawai* Nomura**
4	Metafemur with large cavity and mushroom-like projection in cavity (Yin et al. 2017: figs 1B, 3C) (Shanghai, Zhejiang)	***P.predator* (Sharp)**
–	Metafemur swollen, lacking cavity or projection	**5**
5	Body size larger, length approximately 2 mm; pronotum with short median longitudinal sulcus not reaching anterior margin of pronotum; metafemur with oblique furrow on lateral side (Taiwan)	***P.cruralis* (Raffray)**
–	Body size smaller, length 1.5–1.7 mm; pronotum with long median longitudinal sulcus almost reaching anterior margin of pronotum; metafemur with transverse furrow on upper side (Taiwan; Ryukyu Islands)	***P.schenklingi* (Raffray)**

## Supplementary Material

XML Treatment for Physomerinus jiulongensis
